# The therapeutic potential of inhibiting PPARγ phosphorylation to treat type 2 diabetes

**DOI:** 10.1016/j.jbc.2021.101030

**Published:** 2021-07-31

**Authors:** Rebecca L. Frkic, Katharina Richter, John B. Bruning

**Affiliations:** 1 The Institute for Photonics and Advanced Sensing, and School of Biological Sciences, The University of Adelaide, Adelaide, South Australia, Australia; 2 Richter Lab, Department of Surgery, Basil Hetzel Institute for Translational Health Research, The University of Adelaide, Adelaide, South Australia, Australia

**Keywords:** T2DM, PPARγ, phosphorylation, drug design, ERK, extracellular signal-regulated kinase, LBD, ligand-binding domain, MD, molecular dynamics, PPARγ, peroxisome proliferator-activated receptor γ, PPRE, peroxisome proliferator response element, PTM, posttranslational modification, RXRα, retinoid X receptor α, T2DM, type 2 diabetes mellitus, TZD, thiazolidinedione

## Abstract

A promising approach for treating type 2 diabetes mellitus (T2DM) is to target the Peroxisome Proliferator-Activated Receptor γ (PPARγ) transcription factor, which regulates the expression of proteins critical for T2DM. Mechanisms involved in PPARγ signaling are poorly understood, yet globally increasing T2DM prevalence demands improvements in drug design. Synthetic, nonactivating PPARγ ligands can abolish the phosphorylation of PPARγ at Ser273, a posttranslational modification correlated with obesity and insulin resistance. It is not understood how these ligands prevent phosphorylation, and the lack of experimental mechanistic information can be attributed to previous ambiguity in the field as well as to limitations in experimental approaches; *in silico* modeling currently provides the only insight into how ligands block Ser273 phosphorylation. The future availability of experimental evidence is critical for clarifying the mechanism by which ligands prevent phosphorylation and should be the priority of future T2DM-focused research. Following this, the properties of ligands that enable them to block phosphorylation can be improved upon to generate ligands tailored for blocking phosphorylation and therefore restoring insulin sensitivity. This would represent a significant step forward for treating T2DM. This review summarizes current knowledge of the roles of PPARγ in T2DM as well as the effects of synthetic ligands on the modulation of these roles. We hypothesize potential factors that contribute to the reduction in recent developments and summarize what has currently been done to shed light on this critical field of research.

## PPARγ is a target for treating type 2 diabetes

The Peroxisome Proliferator-Activated Receptors (PPARs) are ligand-modulated transcription factors belonging to the nuclear receptor superfamily of proteins and have critical roles in controlling many processes in the human body. The PPARs are sorted into three subtypes: PPARα, PPARβ/δ, and PPARγ. They differ in their ligand specificity, transcriptional activity, and tissue expression profiles. Of the three subtypes, PPARγ has attracted the greatest research efforts for its roles in a number of diseases (1, 2), and exists in two isoforms; PPARγ1 and PPARγ2, which differ by an additional 30 amino acids at the N-terminus of PPARγ2 (3, 4, 5, 6, 7). The residue numbering convention differs between the two isoforms; for this review the numbering convention for PPARγ2 will be used.

PPARγ binds to a diverse range of endogenous ligands, which act as indicators of the cell’s metabolic state. Endogenous ligands of PPARγ bind to the ligand-binding domain (LBD) of the protein and include metabolites, such as fatty acids and eicosanoids (8, 9, 10, 11, 12), which act through PPARγ to regulate over 100 genes (13) to maintain metabolic homeostasis in the cell. Upon ligand binding, PPARγ forms a heterodimer with Retinoid X Receptor α (RXRα) and binds to the peroxisome proliferator response element (PPRE), a short sequence of DNA located throughout the genome upstream of genes under the transcriptional control of PPARγ (14, 15).

Coregulatory proteins are recruited to the PPARγ/RXRα complex to either transcribe or repress the gene downstream of the PPRE. These large coregulatory proteins recruit chromatin remodelers and are classed as either coactivators, *e.g.*, Steroid Receptor Coactivator (SRC) or Nuclear Receptor Coactivating Proteins (NCoA), or corepressors, such as Nuclear Receptor Corepressor (NCoR) or Silencing Mediator of Retinoic Acid and Thyroid Hormone Receptor (SMRT) (16). Coactivator recruitment unwinds chromatin to expose the target gene, providing access for the transcriptional machinery to promote transcription. Conversely, corepressors maintain chromatin in the repressive, tightly wound state to prevent transcription of the gene. These coregulatory proteins can also act through modulating interactions with transcriptional machinery, such as polymerases (17).

Many different genes are under the transcriptional control of PPARγ (9), which acts as the master regulator to ensure these genes operate in harmony to maintain metabolic homeostasis. These include genes involved in adipogenesis, inflammation, oesteogenesis, immunity, and glucose homeostasis. Because of these critical roles played by PPARγ, a large effort has been invested in developing synthetic ligands specific to PPARγ, which can reverse various disease states, including type 2 diabetes, inflammation (18, 19, 20, 21), and cancer (22, 23, 24, 25). This review will focus on the clinical application of modulating PPARγ to treat type 2 diabetes.

PPARγ activity is vital to the development and prevalence of type 2 diabetes mellitus (T2DM) due to its various roles in metabolism, particularly glucose homeostasis, which is dysregulated in T2DM. One of the main functions of PPARγ is to control adipogenesis in white adipose tissue; in fact, PPARγ is both necessary and sufficient to drive fibroblastic precursors into adipocytes (5, 6). Adipose tissues secrete various cytokines and adipokines, such as adiponectin, leptin, TNF-α, IL-6, and resistin. These proinflammatory molecules have significant effects on insulin resistance and the development of obesity (26). The roles of PPARγ are most prominent in white adipose tissue where the receptor is most heavily expressed, but it is also expressed in the liver and muscle, where it has a direct impact on insulin sensitivity. These critical roles of PPARγ in the development of T2DM make PPARγ an attractive drug target for the treatment and management of T2DM.

**Figure 1:**
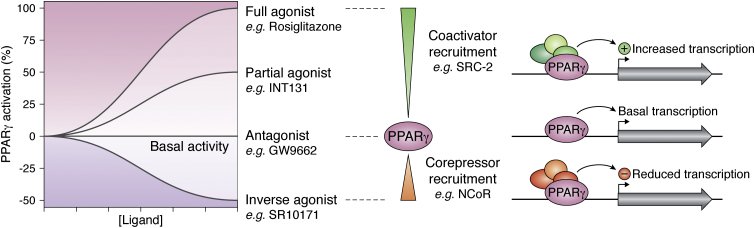
**Ligands of PPARγ cover a spectrum of transcriptional effects.** In the absence of a ligand, PPARγ has a basal level of expression of the genes it controls, and ligands can affect the transcriptional output. This is mediated by the recruitment of transcriptionally promoting coactivators or transcriptionally-suppressing corepressors to PPARγ.

In the absence of a ligand, PPARγ-controlled genes have a basal level of expression and this level can be altered by synthetic ligands binding to PPARγ (Fig. 1). Full agonist ligands systematically turn on genes under the transcriptional control of PPARγ by causing subtle structural changes in the protein to promote coactivator binding. Partial agonists exhibit a similar effect but to a lesser degree, which somewhat promotes coactivator binding to turn on some genes above basal levels. Nonactivating ligands, such as antagonists or inverse agonists, modulate PPARγ by binding with high affinity but without increasing gene expression levels. In the case of inverse agonists, they modulate PPARγ by reducing basal gene expression through the recruitment of corepressors.

A well-studied full agonist of PPARγ is Rosiglitazone (Avandia), an effective insulin sensitizer that was generously prescribed to treat T2DM; yearly sales of Avandia reached US $3.3 billion in 2006 (27), until it was withdrawn from clinical use due to a number of harmful side effects (28, 29, 30). Rosiglitazone is a member of the thiazolidinedione (TZD) class of compounds, which were synthetically designed to stabilize the coactivator-binding surface of PPARγ to strongly recruit transcriptionally promoting coactivators. This leads to a systematic upregulation of PPARγ-controlled genes, which dysregulates normal physiological processes and manifests as harmful side effects such as weight gain, fluid retention, loss of bone density, and congestive heart failure (31, 32, 33). Despite these problematic side effects, the effectiveness of Rosiglitazone as an insulin sensitizer warrants continued efforts into developing PPARγ-targeting antidiabetic ligands.

In light of this, research attention was turned to partial agonists of PPARγ, which showed fewer side effects compared with TZDs (9). Despite their lowered activation of the receptor, partial agonists of PPARγ still displayed insulin-sensitizing properties comparable to Rosiglitazone (34, 35). However, these compounds still upregulate PPARγ-controlled genes above normal levels, and some have displayed a suboptimal pharmacokinetic profile such as partitioning to the liver (36). Researchers have observed that the harmful side effects were associated with upregulation of PPARγ-controlled gene activity, dysregulating the homeostasis maintained by normal PPARγ function (9). Therefore, nonactivating ligands became the focus of PPARγ-targeted antidiabetic therapeutics in recent years. Antagonists and inverse agonists of PPARγ have shown the most promise for treating T2DM for their capacity to normalize insulin sensitivity but without the side effects seen for full or partial agonists. They have been extensively characterized in structure and biochemical properties (37, 38, 39, 40, 41, 42) and represent a very promising avenue for reducing the global incidence of T2DM.

It is apparent from research undertaken with full agonists, partial agonists, and nonactivating ligands of PPARγ that the insulin-sensitizing properties are consistent across these classes of compounds, irrespective of their level of transcriptional effects on the receptor (43). This suggests that their capacity to reverse T2DM is independent of, and can be decoupled from, their transactivation of PPARγ-controlled genes. This is an opportunity to cultivate the insulin-sensitizing effects of these compounds without the risk of side effects and is a fundamental concept that should direct future PPARγ-focused research. This avenue has been opened by recent advances in the understanding of nonactivating ligands with reduced side effects (37, 44, 45, 46). Successfully harboring the insulin-sensitizing effects of these ligands could signify a huge leap in the development of safe and effective therapeutics to reduce the global financial and medical burden inflicted on hundreds of millions of people as a result of T2DM. In order for this to occur, it is imperative that a better understanding of PPARγ is developed.

## Phosphorylation of PPARγ is correlated with insulin resistance

Posttranslational modifications (PTMs) of PPARγ, such as phosphorylation, deacetylation, ubiquitination, and SUMOylation, are adding to the complexity of its modulation (47). Of significant clinical relevance is the phosphorylation of the receptor at specific sites to effect modulation of various processes. A recently identified PTM of PPARγ is phosphorylation at Tyr78, which regulates the expression of cytokines and chemokines and has roles in adipocyte inflammation (48). Ser112 of PPARγ is phosphorylated by mitogen-activated protein kinase (MAPK); its phosphorylation suppresses PPARγ transcriptional activity by inhibiting ligand binding and regulating both the recruitment of corepressors and release of coactivators (48, 49).

**Figure 2:**
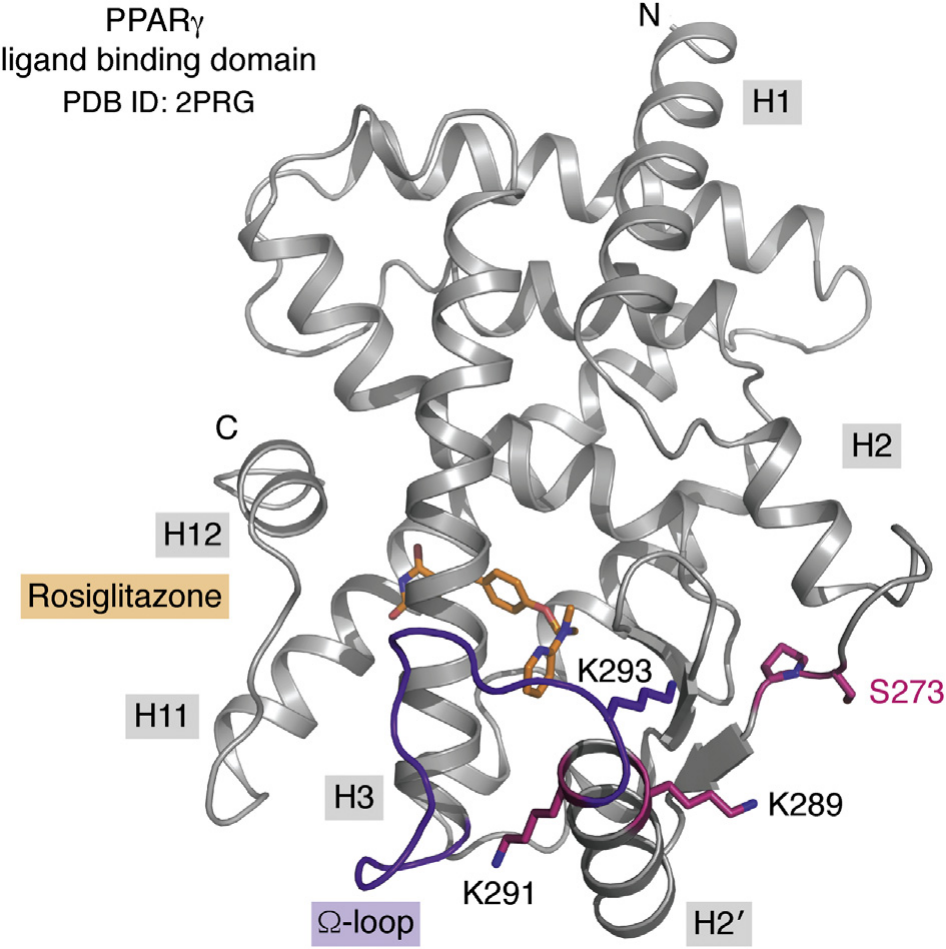
**The ligand-binding domain (LBD) of PPARγ binds to hydrophobic endogenous or synthetic ligands and contains Ser273, a critical regulatory residue that can be phosphorylated.** PPARγ LBD is shown in *gray ribbons*, with Ser273 and other key residues shown in *pink sticks*. The receptor is complexed with Rosiglitazone (*orange sticks*). The Ω-loop, critical for interacting with kinases, is highlighted in *purple* (PDB ID: 2PRG) (51).

The PPARγ phosphorylation site that is of greatest clinical relevance is at Ser273 (Ser245 in PPARγ isoform 1 nomenclature), located within the LBD of PPARγ (Fig. 2). Critically, high levels of phosphorylated Ser273 (pSer273) are observed in obesity and insulin resistance (43, 50). This PTM is key for treating T2DM, as the phosphorylation status of Ser273 appears be a key contributor to the onset of T2DM.

There is a well-studied correlation between high levels of PPARγ phosphorylated at Ser273 with increased obesity and insulin resistance. In order to investigate the mechanism behind this, a fibroblast cell line harboring PPARγ ΔS273A was established as a useful tool for understanding the effects of abolishing Ser273 phosphorylation (51). The authors utilized this mutant to investigate the downstream transcription of a set of genes hypothesized to have roles in the onset of T2DM. Some noteworthy genes whose transcription was increased for the ΔS273A mutant protein include: i) CD36, a membrane protein involved in fatty acid uptake into muscle and adipose tissue (52); ii) adiponectin, a hormone that plays crucial roles in preventing insulin resistance and T2DM (53); iii) adipsin, an adipokine that improves the function of insulin-producing β-cells (54); and iv) leptin, an appetite suppressant (55). These genes had lower levels of expression for wild-type PPARγ, which was capable of being phosphorylated at Ser273, indicating that diminished expression of these genes contributes to the development of T2DM.

The link between the phosphorylation of Ser273 of PPARγ and the differential gene expression leading to the development of T2DM was previously elusive until a 2014 study by Choi *et al.*, (56) which identified a key mechanism connecting the two events. It was discovered that thyroid hormone receptor-associated protein 3 (Thrap3) recognizes and selectively binds to PPARγ when it is phosphorylated at Ser273. Upon binding to PPARγ, this coregulatory protein causes differential expression of a set of genes that promotes insulin resistance, which can lead to the development of T2DM (56). In particular, genes whose transcription supresses insulin resistance and T2DM, such as CD36, adiponectin, adipsin, and leptin, are downregulated by Thrap3 binding to pPPARγ. The authors found that knockdown of Thrap3 led to restored glucose tolerance in high-fat diet fed mice, supporting their model of Thrap3’s role in insulin resistance. Upon further research investment, some key knowledge gaps could be filled such as how Thrap3 causes differential gene expression and whether this is through the transcriptional activities of PPARγ, or if Thrap3 interacts with other downstream proteins to have an effect on gene transcription.

The link between Ser273 phosphorylation and T2DM was built on recently in a mouse model harboring a ΔS273A double allele mutation, serving as an animal model for investigating the effects of inhibiting pSer273 (57). Using PPARγ^S273A/S273A^ mice specifically bred for this study, the authors verified that PPARγ^S273A/S273A^ showed greater insulin sensitivity than wild type when mice were on a high-fat diet. The major finding of their study was that knockout of pSer273 resulted in significant downregulation of Gdf3 mRNA, a member of the TGF-β superfamily of proteins. This suggests that phosphorylation of PPARγ at Ser273 increases expression of Gdf3. Gdf3 expression can affect cellular differentiation, obesity, glucose uptake, and insulin resistance (57, 58, 59, 60). The authors postulate that the roles of Gdf3 in T2DM progression could present as a putative target for reducing the incidence of T2DM.

It is likely that a combination of Thrap3 and Gdf3 contributes to the development of T2DM when PPARγ is phosphorylated at Ser273. Though a direct link between pPPARγ and Gdf3 expression is still unconfirmed, it has been observed that pPPARγ directly interacts with Thrap3 (56). Therefore, it is likely that Thrap3 mediates the effect of pPPARγ on Gdf3 mRNA levels, possibly by acting as a coregulatory protein bound to pPPARγ to transcribe Gdf3, or upon activation by pPPARγ, Thrap3 acts as a coregulatory protein elsewhere to transcribe Gdf3. Given the recent nature of these discoveries, it cannot be ruled out that there are more, not yet identified, proteins involved in this pathway. It is important that this pathway is better understood so that effective insulin sensitizers can be developed.

**Figure 3:**
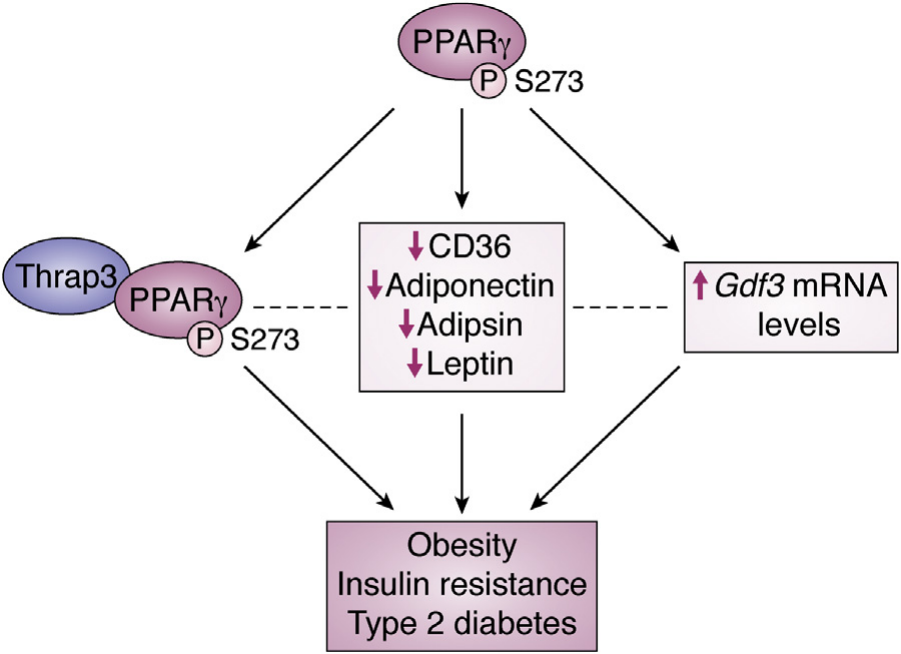
**Current knowledge of how phosphorylation of PPARγ is linked to T2DM.** Phosphorylated PPARγ has been linked to T2DM in a number of studies, which have identified different events in response to this PTM to PPARγ. These include Thrap3 binding to PPARγ (57), decreased expression of CD36, Adiponectin, Adipsin, and Leptin (52), and increased Gdf3 mRNA levels (58). The effect of these pathways on each other is unknown (*dotted lines*), but in each case the pathways lead to obesity, insulin resistance, and type 2 diabetes.

We have summarized the different known pathways that link PPARγ phosphorylation to T2DM (Fig. 3). These pathways may work in symphony together, or they could be overlapping, redundant pathways. Research efforts should be invested to decipher this network of pathways to better elucidate the mechanisms linking PPARγ to T2DM.

## Kinase ambiguity has been hampering pSer273-directed research

The identity of the kinase responsible for phosphorylating Ser273 was difficult to identify. Initially, it was found that cyclin-dependant kinase 5 (Cdk5) catalyzes this PTM (51), and more recently it has been shown that Extracellular Signal-Regulated Kinase (ERK), negatively regulated by Cdk5, is the kinase of interest (50). This ambiguity might have been affecting the productivity of research in this area due to a lack of consensus in the field, and so research has not been unified in its direction.

Cdk5 was the initial candidate for phosphorylating Ser273 of PPARγ based on the observation that in obesity, increased expression of Cdk5 was observed in adipose tissue, as well as higher levels of pPPARγ (51). This was supported by the protein sequence and structure of PPARγ qualifying as a substrate for Cdk5. Cdk5 adopts PPARγ as a substrate by recognizing Ser273 (P0) immediately preceding a proline residue (P + 1). Higher specificity is achieved between Cdk5 and its substrates through a P + 3 positively charged residue (K/R), which is not present in PPARγ but is instead compensated by a structurally close lysine residue (Lys289), forming a noncontiguous motif for Cdk5 interaction (61). Choi *et al.* (51) were able to show *in vitro* phosphorylation of PPARγ incubated with Cdk5 and its cofactor p35 as detected by western blot. Their findings were supported *in vivo*, where they cotransfected 3T3-L1 adipocytes with Cdk5 and wtPPARγ or PPARγ^S273A^ and found that phosphorylation of PPARγ was suppressed by shRNA-mediated knockdown of Cdk5, or by roscovitine, a selective Cdk5 inhibitor. This provided convincing evidence that Cdk5 is able to phosphorylate PPARγ at Ser273.

More recent work by the same group revealed further data, where they observed higher levels of phosphorylated PPARγ and lowered insulin sensitivity in an adipocyte-specific Cdk5-KO mouse model (50). The authors found that in the adipocytes of this Cdk5-KO model, ERK was highly phosphorylated (activated) and was perhaps also, or instead, responsible for this important PTM of PPARγ. The former was confirmed by showing that ERK kinase was also able to phosphorylate Ser273 of PPARγ *in vitro* in the same capacity as in the previous experiments using Cdk5. This was unsurprising, given that ERK is structurally similar to Cdk5 and also acts through a Ser/Thr-Pro recognition motif (62). From these data combined, the authors postulated that Cdk5 might have a regulatory effect on ERK. They identified MEK2, the kinase upstream of ERK in the Grb2/Ras/MEK/ERK kinase cascade, as being phosphorylated by Cdk5 at a novel site, *i.e.*, Thr397. This PTM is unlike the phosphorylation of Ser222 and Ser226 of MEK, which have an activating effect through the capacity to phosphorylate ERK (63, 64, 65, 66). Instead, MEK^pThr397^ is incapable of phosphorylating ERK, thereby repressing PPARγ phosphorylation. From this, it can be postulated that Cdk5 phosphorylates MEK at Thr397, which prevents the phosphorylation of ERK and hence ERK-mediated phosphorylation of PPARγ. On the same note, Cdk5 deletion results in the activation of ERK through the absence of the repressive Thr397 phosphorylation.

**Figure 4:**
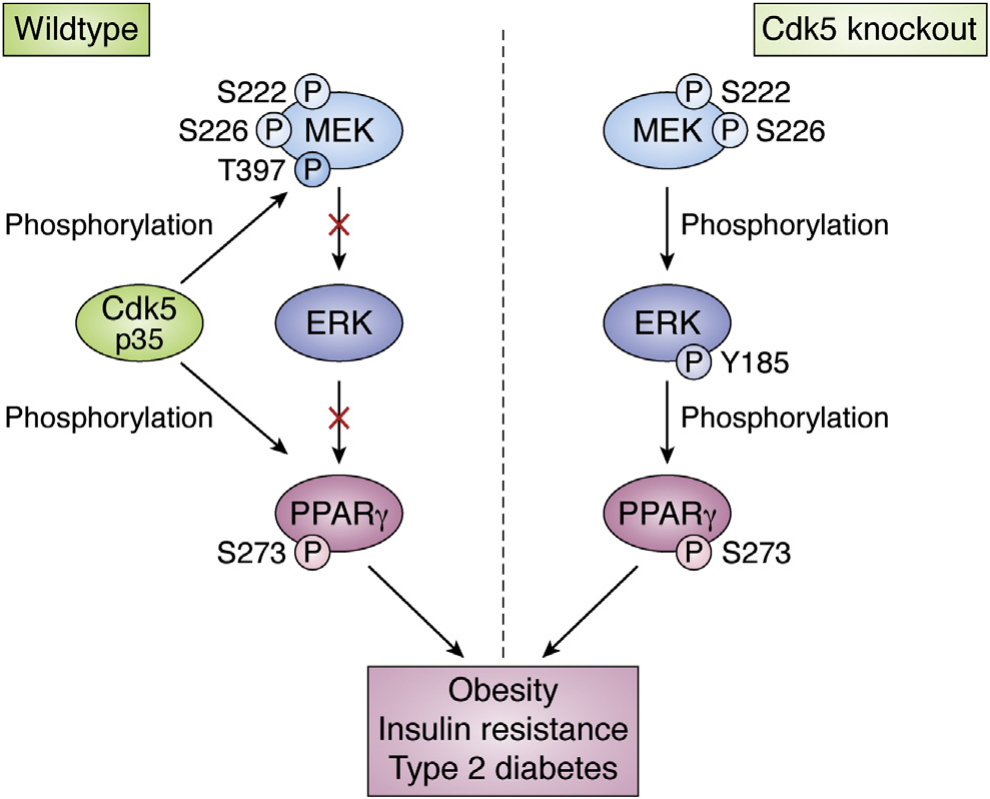
**Redundancy in the PPARγ phosphorylation pathway.** Work by Banks *et al.* (50) showed that in a Cdk5-KO mouse model, phosphorylation of PPARγ is compensated by ERK kinase. Upon phosphorylation of MEK by Cdk5 at Thr397, MEK is unable to phosphorylate ERK, and therefore ERK cannot phosphorylate PPARγ. Both of these pathways result in PPARγ phosphorylation at Ser273, which is implicated in obesity, insulin resistance, and T2DM.

These findings combined can be presented in a simplified model, where there is redundancy in the Cdk5/ERK phosphorylation pathway to achieve the same outcome of phosphorylating PPARγ (Fig. 4). Based on the initial observation by Banks *et al.*, which saw higher pPPARγ in response to Cdk5-KO, it would appear that the MEK-ERK pathway results in a higher population of pPPARγ compared with the combined effect of both kinases.

Although it has been suggested previously that a MEK inhibitor will hold promise as a potential antidiabetic compound (50), this may have limitations when Cdk5 compensates for the loss of MEK/ERK activity. Additionally, targeting the MEK/ERK pathway or the Cdk5 pathway with inhibitors will likely have a devastating long-term effect on an organism due to the myriad of pathways regulated by these kinase signaling cascades.

No additional research has been reported on the role of the Thr397 phosphorylation since its first publication in 2015. There is a general consensus in citing literature that Cdk5 is the sole kinase responsible for phosphorylating Ser273 of PPARγ (44, 45, 46, 61, 67, 68, 69). Recent publications acknowledged the activities of ERK as the kinase of interest (57, 70, 71) or cited a combination of the two kinases (37, 72, 73, 74). It is most likely that both kinases contribute to phosphorylating PPARγ in an organism, and this should be kept in mind when conjecturing selective kinase-specific inhibitors. Clearly, the safest and most effective approach to preventing phosphorylation of Ser273 of PPARγ is through targeting PPARγ itself with synthetic ligands.

## Antidiabetic ligands of PPARγ block Ser273 phosphorylation

A number of PPARγ ligands have been developed to combat T2DM, showing the capacity to normalize insulin sensitivity through blocking Ser273 phosphorylation (51). These ligands are able to restore gene expression levels to a normal, nondiabetic state after being dysregulated by high-fat diet-induced T2DM in mice (75). The ligands of PPARγ capable of blocking Ser273 phosphorylation include full agonist Rosiglitazone (43, 51), as well as some ligands of other transcriptional classes, such as partial agonists (51, 76, 77), antagonists (43), and inverse agonists (42). These ligands show a similar capacity to inhibit Ser273 phosphorylation irrespective of their transcriptional effects, which occur independently of antidiabetic properties. Targeting PPARγ with synthetic ligands assists in improving glucose homeostasis and, in the case of nonactivating ligands of PPARγ, can exert their effects with minimal side effects.

However, there is a lack of experimental, mechanistic information explaining how PPARγ ligands block Ser273 phosphorylation. Addressing this gap in knowledge will enable the efficient development of PPARγ ligands at the molecular level to enhance their capacity to block phosphorylation, which would yield more effective insulin sensitizers.

Research in this field has potentially been hampered by the initial ambiguity surrounding the kinase responsible for phosphorylating Ser273. In addition, crystal structures have been unable to provide insight due to the position of Ser273 in the protein. It is positioned on the H2-H2′ loop (Fig. 2), which is subject to stabilizing crystal contacts in most cases, and where it is not artificially stabilized, it can be disordered enough to warrant not modeling the loop or modeling into ambiguous data. Most PPARγ ligands in crystal structures are bound in the ligand-binding pocket distal to the site of phosphorylation, leaving their roles in blocking phosphorylation unclear. Comparing apo crystal structures, which are amenable to phosphorylation, with ligand-bound PPARγ crystal structures there appears to be minimal structural variation in this region of the protein, suggesting that the aforementioned limitations of crystallography may be hampering this critical discovery.

Circumventing the hurdles to solving the mechanism by which ligands block Ser273 phosphorylation will have considerable payoff in terms of providing information to lead future developments of antidiabetic PPARγ ligands. Effort should be invested into crystallizing the phosphorylated protein or PPARγ in complex with kinase, as this undiscovered knowledge could provide critical insights. For example, revealing any structural accommodations essential for interaction with kinases, which would be prevented by ligand binding.

## *In silico* approaches have provided putative mechanistic information

*In silico* molecular modeling simulations have been performed to shed light on possible structural scenarios determining the phosphorylation of PPARγ by kinases and the impact of ligands on this.

One approach was to use molecular dynamics (MD) simulations to study the conformational dynamics of the full-length PPARγ and full-length RXRα complex bound to DNA (78). Lemkul *et al.* used the crystal structure of full-length PPARγ in complex with full-length RXRα bound to the PPRE DNA sequence solved by Chandra *et al.* (79) and tested different MD scenarios on the complex ± ligand and coactivator peptide, as well as Ser273/pSer273. One of their discoveries was that while the H2′-H3 loop (Ω-loop, refer to Fig. 2) showed a high degree of flexibility in the unphosphorylated state, it was much more tightly associated with the remainder of the LBD when Ser273 was phosphorylated. The authors found this movement in the Ω-loop to propagate through the remainder of the LBD and the entire protein, causing changes in the conformation and dynamics of the PPARγ hinge region as well as the DNA-binding domain. These long-range effects may determine differential transcriptional output through interaction with coregulators as well as with DNA and with RXRα. Importantly, the authors note that their ligand of choice, selective PPARγ partial agonist BVT.13 (80), samples different conformations within the large ligand-binding pocket of PPARγ. The distribution between these conformations was affected by the phosphorylation state of PPARγ in their simulations. It was hypothesized that this may allow for the binding of specific coactivators, which can lead to different genes being turned on or off. This aligns with the finding that Thrap3 binds to pPPARγ, which could be facilitated by phosphorylation-specific stabilization of pPPARγ.

Another *in silico* approach was to use crystal structures of PPARγ LBD and Cdk5/p25 kinase to predict the binding interaction between the two proteins (81). The interaction between PPARγ and Cdk5 is extensive in their model, with the Ω-loop, the β-sheet, and the Ser273 loop of PPARγ participating in interactions. The authors noted that in their model, the Ω-loop turns out from the remainder of the LBD, exposing residues critical to forming interactions with Cdk5. This is in contrast to ligand-bound crystal structures of PPARγ LBD, where the Ω-loop is closely associated with the LBD, usually stabilized by interactions with the ligand. This indicates that a conformational change is required in the Ω-loop of PPARγ in order to allow binding to Cdk5, which brings the enzyme within close proximity of Ser273 to catalyze phosphorylation of this residue. This hypothesis is plausible given the dynamic nature of this loop and its apparent importance in interacting with Cdk5. However, a complicating factor for experimentally validating this hypothesis is the dynamic nature of the Ω-loop, resulting in poor data quality for this region in crystal structures, making it difficult to draw conclusions based on the modeled conformation of this stretch of residues. In fact, it is quite common for crystallographers to refrain from modeling any residues for this loop due to ambiguity in the electron density. This hurdle could be overcome by cocrystallizing the PPARγ LBD-Cdk5/p25 complex, which should stabilize the Ω-loop as well as confirm the binding model proposed by Mottin *et al.*

Similar to Mottin *et al.*, another research group used protein–protein docking to investigate the key residues required for Cdk5/PPARγ interaction (61). They superimposed binding modes of Cdk5 as well as ERK to PPARγ to identify the conserved interacting residues between the two kinases. Two of the conserved residues from the kinases (Asp40/Glu60 and Glu194/Glu220) form critical interactions with three lysines in PPARγ, *i.e.*, Lys268, Lys291, and Lys293 (Lys240, Lys263, and Lys265 in PPARγ1 isoform nomenclature) (Fig. 2). The authors focused on these lysine residues as well as Lys289 (Lys261 in PPARγ1 isoform nomenclature), which is important for spatially forming the P + 3 residue for recognition by Cdk5. They individually mutated each lysine to an alanine, which showed a reduction in pSer273 in each case except for Lys268, suggesting that the others are critical to enable interaction with Cdk5 to phosphorylate PPARγ. Interestingly, PPARγ ligands do not interact with or otherwise stabilize these lysines. The authors postulated that ligands capable of blocking Ser273 phosphorylation do so by causing a shift in the orientation of Ile369, which reorients to complete a stabilizing hydrophobic network comprised of Met376 (β-sheet), Ile277 (H2-H2′ loop), and Leu283 (H2′), to stabilize regions of the LBD to make them unavailable for interaction with kinase. These regions that are stabilized include the residues Lys291 and Lys293, which form electrostatic interactions with Cdk5, as well as Lys289, which forms the P + 3 residue in the Cdk5 recognition motif (refer to Fig. 2). This hypothesis is similar to previous analyses, which attributed the capacity of the ligands to block phosphorylation to their ability to create a stabilizing environment within the LBD to discourage interaction with kinases (81). This was supported experimentally by Ribeiro Filho *et al.* (61), who found that rosiglitazone-bound PPARγ was unable to bind to Cdk5, suggesting that PPARγ ligands prevent PPARγ interaction with the kinase.

Another group also sought to use protein–protein docking to extract information that may be helpful in deciphering the mechanism of ligands blocking phosphorylation (82). The major finding was that a partial distortion of the β-4 strand caused PPARγ to be more amenable to phosphorylation and that ligands of PPARγ served to stabilize this β-strand, thereby exerting their inhibitory effects on phosphorylation. The authors extended their approach to experimental methods to validate the discovery. They used mutagenesis to incorporate two cysteine residues among the β-sheet region to form a disulphide bond that would serve to stabilize the β-4 strand and saw that this construct was not able to be phosphorylated, even in the absence of ligands. In addition, the authors compared various binding poses of PPARγ ligands and observed a general trend in the level of stabilization of the β-4 strand with inhibition of phosphorylation. Based on NMR, they postulated a long-range cross talk mechanism of ligands stabilizing the β-4 strand, mediated by Ile309 on helix 3. This stabilization of the β-4 strand was exerted through Met376, a similar finding to Ribeiro Filho *et al.* (61), who noted the importance of Met376 in forming a stabilizing hydrophobic pocket at this position in the LDB.

In each of the attempts to characterize the mechanism of ligands preventing phosphorylation of Ser273, a consensus conclusion was drawn, which is that ligands binding to PPARγ stabilize regions of the LBD to make them less available for energetically plausible binding with a kinase.

## Conclusion

There are a number of challenges that have been hampering the development of effective antidiabetic ligands of PPARγ, and research efforts should focus on overcoming them to advance understanding of PPARγ modulation and signaling. An example of these challenges is the ambiguity of the kinase responsible for phosphorylating PPARγ, which is potentially causing confusion and lessened productivity in this field. The apparent complexity of the signaling pathways involved in T2DM is also a challenge to the development of safe and effective inhibitors of PPARγ phosphorylation. Some knockout studies have provided some surprising results, which have, in some cases, raised more questions than they have answered, and this level of complexity could likely take decades to elucidate in order to overcome this challenge. Given the increasing incidence of T2DM, it is imperative that these challenges are overcome and research efforts continue to develop understanding of the mechanism of blocking phosphorylation by antidiabetic ligands.

The failure of TZDs as a result of poor mechanistic understanding highlights how critical it is to understand molecular mechanisms of potential drugs before they progress through the drug discovery pipeline. The effectiveness of TZDs and other PPARγ-targeting compounds at restoring insulin sensitivity certainly merits continued research efforts in the development of these compounds. This is especially in light of the advantages of nonactivating ligands of PPARγ, which maintain the capacity to block phosphorylation independently of transcriptional activation.

It is critical to understand how ligands of PPARγ block Ser273 phosphorylation so that future drug design efforts can harbor this capacity and optimize compounds at the molecular level to yield drugs that will be very effective at blocking this phosphorylation. It is already understood that increased transactivation of PPARγ will result in dysregulated gene activation, which manifests as side effects and so drug design efforts have focused on reduced transcriptional capacity in the form of antagonists and inverse agonists. Improvements have been made in the affinity, potency, and pharmacokinetic profile of developing PPARγ ligands. The remaining property of these compounds left to understand and optimize is perhaps the most important one—their mechanism of restoring insulin sensitivity.

Current research efforts indicate that ligands of PPARγ are not in direct contact with the site of phosphorylation, or with the kinase, but instead cause subtle structural changes in the global dynamics of PPARγ to make the protein less favorable to interaction with kinases. This is most likely achieved by ligands closely associating with residues comprising the ligand-binding pocket, including the Ω-loop, to make them unavailable for interaction with kinases, which require flexibility in this loop to achieve an induced-fit mechanism of binding to PPARγ. This has been exhibited by predominantly computational methods, and research should focus on experimentally validating this model to develop a well-established mechanism of blocking phosphorylation by ligands. Successful completion of this will yield safe and effective PPARγ ligands that will increase insulin sensitivity in patients by blocking the phosphorylation of Ser273.

It is critical for the final piece of the puzzle—experimental evidence for the mechanism of how PPARγ ligands block Ser273 phosphorylation—to be robustly investigated in order to inform future drug design efforts to lower the incidence of T2DM.

## Conflict of interest

The authors declare no conflicts of interest.
